# Tansy in Pruritis Vulvæ

**Published:** 1878-12

**Authors:** 


Tansy in Pruritis Vulvæ—Dr. Richard L. Butt, of Midway, Ala., extols the use of tansy (tanacetum hortense) for the relief of pruritis vulvæ. He has found a poultice made of the leaves of the plant, and applied as hot as the patient can bear it, to be efficacious when leeches to the thighs, washes of borax, lead, zinc, nitrate of silver, sulphate of copper, etc., had been tried in vain. The editors of the *American Practitioner*, which records the above, thinks that possibly the mode of using the tansy, in poultice as hot as can be borne, has something to do with the success which has attended the treatment in the hands of Dr. Butt.—*London Lancet.*

